# Why nature prevails over nurture in the making of the elite athlete

**DOI:** 10.1186/s12864-017-4190-8

**Published:** 2017-11-14

**Authors:** Evelina Georgiades, Vassilis Klissouras, Jamie Baulch, Guan Wang, Yannis Pitsiladis

**Affiliations:** 1 Cyprus Sport Organisation, Nicosia, Cyprus; 2 Department of Sports Medicine and Biology of Physical Activity, National and Kapodistrian University of Athens, Greece University of Athens, Athens, Greece; 3 World Champion (4x400m), Cardiff, UK; 4 Centre of Sports Medicine for Anti-Doping Research, University of Brighton, 30 Carlisle Road, Eastbourne, BN20 7SN UK; 5 Department of Movement, Human and Health Sciences, University of Rome “Foro Italico”, Rome, Italy

**Keywords:** Nature, Nurture, Genes, Twin studies, Heritability, Trainability, Sport performance

## Abstract

While the influence of nature (genes) and nurture (environment) on elite sporting performance remains difficult to precisely determine, the dismissal of either as a contributing factor to performance is unwarranted. It is accepted that a complex interaction of a combination of innumerable factors may mold a talented athlete into a champion. The prevailing view today is that understanding elite human performance will require the deciphering of two major sources of individual differences, genes and the environment. It is widely accepted that superior performers are endowed with a high genetic potential actualised through hard and prodigious effort. Heritability studies using the twin model have provided the basis to disentangle genetic and environmental factors that contribute to complex human traits and have paved the way to the detection of specific genes for elite sport performance. Yet, the heritability for most phenotypes essential to elite human performance is above 50% but below 100%, meaning that the environment is also important. Furthermore, individual differences can potentially also be explained not only by the impact of DNA sequence variation on biology and behaviour, but also by the effects of epigenetic changes which affect phenotype by modifying gene expression. Despite this complexity, the overwhelming and accumulating evidence, amounted through experimental research spanning almost two centuries, tips the balance in favour of nature in the “nature” and “nurture” debate. In other words, truly elite-level athletes are built – but only from those born with innate ability.

## Background

The making of champions and achieving elite performance in sport have long been the subject of intense debate – from both a theoretical and a practical perspective. The “nature” versus “nurture” debate dates as far back as the fifth century BC, with one of the first known accounts for the relative nature versus nurture contribution to health and “regimen” having been presented by Hippocrates (460–370 BC), universally honored as the father of medicine, In his Book 1 *Περί Διαίτης (Dietetics)* he noted:

“*Eating alone will not keep a man well; he must also take exercise. For food and exercise, while possessing opposite properties, yet contribute mutually to maintain health. For it is the nature of exercise to use up material, while of food and drink to restore them. And it is necessary, as it appears, to determine exactly the powers of various exercises, both natural exercises and artificial, and which of them contribute to the development of muscle and which to wear and tear; and not only this, but also to proportion exercise to the quantity of food, to the predisposition of the person, to his age, to the season of the year, to the changes of the winds, to the geographical place in which the person resides, and to the climatological conditions of the specific year*”.

Hippocrates made reference not only to nurture (to include foods and exercise, in addition to other significant environmental conditions) as a requisite for *positive health*, but also to an individual’s “genetic predisposition” – in other words, heritability.

Centuries later, Galton (who conceived standard deviation as the measure to quantify normal variation) seemed to be the first academic to give an opinion as to which is more important, with the Galtonian model advocating a hereditary ceiling to physical and mental capacities [1, 2] and objecting “pretensions of natural equality”. This implied that performance is limited by heritable characteristics, which are insurmountable by any amount of practice and training. In his landmark paper “The history of twins as a criterion of the relative powers of nature and nurture” Francis Galton used the following eloquent parable to illustrate the notion of the preponderance of nature on phenotypic variation [3]:

“*Many a person has amused himself by throwing bits of stick into a tiny brook and watching their progress; how they are arrested, first by one chance obstacle, then by another and again, how their onward course is facilitated by a combination of circumstances. He might ascribe much importance to each of these events, and think how largely the destiny of the stick had been governed by a series of trifling accidents. Nevertheless, all the sticks succeed in passing down the current, and in the long run, they travel at nearly the same rate. The one element that varies in different individuals, but is constant in each of them, is the natural tendency; it corresponds to the current in the stream, and inevitably asserts itself...There is no escape from the conclusion that Nature prevails enormously over Nurture*”.

If one accepts that differences between elite and less accomplished performers reflect inherent abilities (so-called “talent”), then it is reasonable to assume that any improvement in performance beyond a fixed maximal level is unmodifiable by extrinsic environmental factors. Empirical evidence has repeatedly refuted this assumption across a wide and diverse range of attributes, including physical performance and motor skills. In particular, improved performance in sport – evidenced by faster times on the track and greater distances in the field events as measured and recorded under strict standardised conditions at national and world level – has clearly been demonstrated despite some leveling off of these increases in some events. However, these improvements in sporting performance over the years can adequately be explained by increases in the duration and intensity of training, new training methods, and even changes to equipment and rules. A recent example of the contribution of extrinsic factors other than nature to performance improvement is the SUB2 marathon project (www.sub2hrs.com); the first dedicated international research initiative made up of specialist multidisciplinary scientists from academia, elite athletes and strategic industry partners with the aim to promote high performance marathon running without doping. While there are no guarantees the SUB2 marathon project will succeed in delivering a 1:59:59 marathon within 5 years, the SUB2 team boast a 100% marathon success record in 2016 and the second fastest marathon in history at the 2016 Berlin Marathon with a time of 2:03:03.

The theoretical framework for “*deliberate practice*”, on the other hand, presents this idea as the means to expert performance and limits the role of innate/inherited characteristics on optimal performance. Ericsson et al. [4] argued that commitment to deliberate practice and effort, over a specific period of time, is the distinguishing factor between the qualitative differences that exist between expert and normal performance. Elite performance, they claim, is the “product of a decade or more of maximal efforts to improve performance in a domain through an optimal distribution of deliberate practice”, thus rejecting the Galtonian model of innate ability in the making of champions. Domain-specific talent – especially when identified at a young age – is perceived as supporting and motivating early practice and attainment of high levels of deliberate practice, eventually resulting in elite performance. This is in stark contrast to the notion that talent in itself reflects inherent exceptional abilities. Ericsson went on to further develop his model, proposing a specific volume of 10,000 h of training to be accumulated over a period of approximately 10 years, as necessary for achieving expert levels [5]. Despite the widespread appeal and popularity of Ericsson’s idea as reflected in the emergence of a string of popular books such as Outliers [6], The genius in all of us [7], Bounce [8], the evidence to support a key role for longstanding practice is lacking. To proclaim that “genetic talent” is just a myth, therefore, seems misguided. The most compelling opposition to Ericsson’s idea is the finding that performance is poorly related to deliberate practice time. For example, only 28% of the variance in performance in the sport of darts could be explained by accumulated training [9]. The theory that performance is constrained by accumulated hours of deliberate practice is also further weakened by studies showing that elite athletes rarely complete the necessary 10,000 h of training before reaching world-class level [10, 11]. The lack of measures of variance (standard deviation or ranges) presented by Ericsson et al. [4] also significantly weakens their argument for an association between training and performance, as applicable to every individual. A number of studies examining the relationship between training and performance especially of skill-based activities, revealed significant individual variation as reflected by a large standard deviation and coefficient of variation [12, 13]. For example, Gobet and Campilelli [13] investigated markers of talent (e.g., handedness), the environment, and the critical period for the acquisition of expert performance in Argentinian chess players (*N* = 104), ranging from weak amateurs to grandmasters. Their findings reaffirmed the importance of practice for the attainment of high levels of performance but also revealed large variability. Notably, some players needed 8 times as much practice to reach master level than others. The authors concluded that practice was necessary, but not a sufficient condition for the acquisition of expertise and that some additional factors seemed to differentiate chessplayers and non-chessplayers. In terms of studies involving sporting domains, Hodge and Deakin’s [14] investigation of karate experts and novices is characteristic of the literature, revealing no differences in the total number of hours per week spent participating in karate-related activities at the beginning of the athletes’ careers (e.g., novice: 35.3 h/week; expert: 27.6 h/week). Neither did these authors find differences in the amount of time engaged in the activities considered most relevant to martial arts performance such as sparring, classes, impact training and kata training alone and with others (e.g., novice: 12.8 h/week; expert: 13.7 h/week). Despite similarities reported in time spent in deliberate practice in these martial arts athletes with deliberate practice across other sporting domains (e.g., wrestlers), these results overall did not support the idea that individual differences in expert performance reflect largely individual differences in the amount of deliberate practice. This conclusion echoes the findings of a recent meta-analysis of deliberate practice and performance involving 88 eligible studies [15], which found that deliberate practice explained 18% of the variance in performance in sports, 26% for games, 21% for music, only 4% for education and less than 1% for professions and concluded that deliberate practice is important, but not as important as has been previously argued by Ericsson and his colleagues. The authors of this meta-analysis also found the effect of deliberate practice on performance tended to be larger for activities that are highly predictable (e.g., running) than for activities that are less predictable (e.g., fencing). On the basis of the previous literature on deliberate practice, it would appear that additional factors differentiate experts from novices. It is our contention that these additional factors/characteristics, once identified, will emerge to be substantially heritable in nature.

The large variability in all essential attributes and/or responses is precisely what would be predicted and underpins the present day concept of precision/personalised medicine, where major international consortia are attempting to correlate genomic and other high-throughput “omics” data in order to identify individual differences in the response to treatment of major medical conditions such as cancer, and type 2 diabetes. The development of biomarkers for personalised oncology is a striking example of how this large inter-individual variability in response can potentially be harnessed to improve efficacy of treatment. In the last decade there have been significant advances in the development of biomarkers for novel drug targets and new treatment strategies for patients with advanced-stage cancers are moving away from traditional treatment strategies to biomarker driven treatment algorithms based on the molecular profile of each tumor. As such, predictive biomarkers are increasingly being used to match targeted therapies with patients, and prevent toxicity of standard therapies [16].

### The evidence from genomics, genetics and exercise biology

Heritability studies on physical performance and functional adaptability provided strong evidence of a significant genetic component to various parameters that ultimately determine elite performance. Over the past two decades there has been a clear shift in terms of how sports and exercise genetics research has been conducted and the ever-increasing focus on determining specific genes linked to performance. Early family studies/twin models (see Table 1, illustrating key milestones in genomics, genetics and exercise biology over past decades) have provided the basis for disentangling the genetic and environmental factors that contribute to complex human traits, and subsequent genetic association studies in unrelated individuals have further paved the way to detect specific genes for elite sport performance. The pioneering studies on twins [17] revealed that as much as 93% of variability in maximal aerobic capacity ($\overset{\cdotp }{\mathrm{V}}{\mathrm{O}}_2\max$) is genetically determined in 25 pairs of monozygotic (*n* = 15) and dizygotic (*n* = 10) twins. The model developed by Klissouras assumed comparable environmental influences between the two sets of twins and the absence of gene-environment interactions. Klissouras also found that maximal anaerobic capacity and maximal heart rate are heavily dependent on genes, which accounted for 81% and 86% of the variation of traits, respectively. Subsequent studies applying path analysis of twin and nuclear family data also reported a high genetic component for $\overset{\cdotp }{\mathrm{V}}{\mathrm{O}}_2\max$, namely 77% [18], 69% to 87% [19], and 71% [20], while a more recent meta-analysis of eight twin studies generated a weighted $\overset{\cdotp }{\mathrm{V}}{\mathrm{O}}_2\max$ heritability estimate of 72% [21]. Notable exceptions are the lower heritability estimates of 40% and 51%, for $\overset{\cdotp }{\mathrm{V}}{\mathrm{O}}_2\max$ reported by Bouchard and colleagues [22, 23]. Heritability estimates have also been reported for other phenotypic traits linked to sporting performance, such as 99% for maximal anaerobic power [24], 66–92% for muscle cross-sectional area and body dimensions [25, 26], 93–100% for muscle fiber distribution [27], 85% and 73% for neuromuscular coordination at 70% and 50% of maximal velocity, respectively [28], 68% for motor control and motor learning [29], 68% for motor cortex plasticity [30], 80% for intracortical inhibition and 92% for intracortical facilitation [31], 40–50% for personality traits, and 38–71% for specific cognitive abilities [32]. Indeed, in a review by Singer and Janelle [33], cognitive abilities and the overall impact of the brain and how it works, are implied as being of significant importance in the determination of expert performers, compared to novices, in that experts are identified as having, inter alia, the ability to make more rapid and appropriate decisions, to use situational probability information better, and to encode and retrieve relevant information more efficiently. The twin study approach has also demonstrated that the acquisition of motor skills is significantly heritable [29, 34]. For example, Fox and colleagues studied learning in a sample of monozygotic and dizygotic twins who had been reared apart [34]. Specifically, these authors found that heritability of performance at a rotary pursuit task, in which subjects learned to track a rotating target with a stylus, was high even at baseline (66%) and increased with practice (74%), and concluded that the effect of practice is to decrease the effect of environmental variation (previous learning) and increase the relative strength of genetic influences on motor performance. Taken together, virtually all individual differences in functional capacities, morphological dimensions, motor attributes, personality traits and cognitive abilities are moderately to substantially heritable. This is in line with the most comprehensive meta-analysis of virtually all twin studies published in the past 50 years, on a wide range of traits and reporting on more than 14 million twin pairs across 39 countries, that provide compelling evidence that all human traits are heritable [35]. Estimates of heritability clustered strongly within functional domains, with the largest heritability estimates for traits classified under the ophthalmological domain (i.e., 0.712, s.e.m. = 0.041), followed by the ear, nose and throat (i.e., 0.637, s.e.m. = 0.064), dermatological (i.e., 0.604, s.e.m. = 0.043) and skeletal (i.e., 0.591, s.e.m. = 0.018) domains. Substantially lower heritability estimates were reported for traits in the environment, reproduction and social values domains (i.e., 0.290–0.313).

**Table 1:** Some key milestones in genomics, genetics, and exercise biology

1971	Vassilis Klissouras/Twin Studies of $\overset{\cdotp }{\mathrm{V}}{\mathrm{O}}_2\max$ [17]
1984	Claude Bouchard/Twin Studies of trainability of $\overset{\cdotp }{\mathrm{V}}{\mathrm{O}}_2\max$ [43]
1999	Claude Bouchard/Heritage Family Study [44]
2000	Hugh Montgomery/Candidate Gene Approach – *ACE* [49]
2001	The Human Genome Project - Initial sequencing and analysis of the human genome (http://web.ornl.gov/sci/techresources/Human_Genome/index.shtml)
2003	The ENCODE Project – large public research consortium aimed at identifying all functional elements in the human genome sequence (www.encodeproject.org)
2003	Kathy North/*ACTN3* Speed Gene [50]
2007	Yannis Pitsiladis/Genetics of East African Runners [45]
2008	The 1000 Genomes Project – the largest public catalogue containing human variation and genotype data (www.internationalgenome.org)
2016	GAMES/The first GWAS of athletic performance [52]
2016	The Athlome Project – call for international collaborated efforts in genetic discovery for elite human performance, muscle injury prevention and adaptive training [53]

*see Bouchard and Malina, 2014 [66] for a detailed account of the history of genomics, genetics, and exercise biology

As the twin studies/heritability estimate approach has received scathing criticism [36–38], it is helpful to fully explain the concept of heritability, which is often misunderstood. For example, a heritability estimate of 93% for a given trait such as $\overset{\cdotp }{\mathrm{V}}{\mathrm{O}}_2\max$ is often misinterpreted to mean that 93% of this phenotype is genetically determined and the remaining 7% is susceptible to environmental modification. Heritability has no etiologic role in a phenotype, nor is it meaningful in terms of measurement in an individual. It is a statistical measure, expressed as a proportion, and refers only to the population under study. More specifically, it describes the extent to which heredity affects the variation of a given attribute in a given population exposed to common environmental influences at a given time. A high heritable attribute does not mean that a phenotype is predetermined and the environment has no effect. It only indicates that the observed individual differences in the given attribute are due to genetic differences and are highly predictable [39]. A frequently overlooked limitation of the early twin studies is that nearly all heritability estimates have been derived using twins exposed to normal environmental influences and represent the normal range of the bell curve and not elite-level athletic twins. The necessity for a nature-nurture investigation using Olympic twin athletes who have actualised their genetic potential with strenuous athletic training and represent the high end of the distribution is clearly required, in that it may provide new insight and may have far-reaching implications to the nature and nurture debate [40]. Further limitations of research on twins are also addressed by Ericsson [4], who advocates “*twin studies of the acquisition of elite performance are unlikely ever to resolve the issue of heritability of elite performance”*. Despite some valid criticisms, twin studies have been an integral part of science for nearly a century and have enhanced significantly our understanding of the extent to which certain traits are inherited.

Despite the mainly indirect evidence favouring a more prominent role of nature over nurture, deliberate practice and environmental factors are undoubtedly both critical to sporting excellence, but they do not in themselves produce elite athletes. Wang et al. [41] defined world-class performance as “a polygenic, multifactorial trait, determined by the interaction of genes and the environment”. The value of training is by no means refused but rather it is proposed that training is defined as the realisation of one’s genetic potential [42]. One of the authors (JB), who spent two decades training and reached world-class level in the 400 m track event in athletics, strongly advocates the case for inherent talent as a prerequisite for elite performance. Having trained with a large group of athletes, only few went on to reach world- and Olympic-level. When all other extrinsic factors (the nurturers) are consistent – the time spent training, the type of training, the facilities, the training environment – what will ultimately distinguish elite performers is their genetic make-up.

The concept of individual differences in the response to exercise training or trainability was also defined empirically more than three decades ago in a series of experimental studies with pairs of monozygotic twins and evidence reported in support of a strong genotype dependency of the ability to respond to regular exercise [43] (Table 1). In the HERITAGE Family Study that ensued [44], it was observed that the heritability of the $\overset{\cdotp }{\mathrm{V}}{\mathrm{O}}_2\max$ response following 20 weeks of standardised exercise training reached 47% after adjustment for age, sex, baseline $\overset{\cdotp }{\mathrm{V}}{\mathrm{O}}_2\max$ and baseline body mass and composition. Notably, there was 2.5 times more variance in individual differences in training response between families than within families. Neither candidate gene studies nor genome-wide explorations have, to date, yielded any validated gene targets and variants as originally anticipated.

Despite some early progress, the question remains as to which genetic variants are those that irrefutably define elite athletic performance and trainability, as numerous attempts to discover candidate genes have largely proved inconclusive, even when genetic superiority was widely assumed as in the African runners phenomenon [45, 46] or conversely the lack of African-American swimmers excelling on the world stage [47]. This outcome is not surprising, given the complexity of both the genomic and phenotypic features in humans. As of 2008, over 200 genes were associated with human physical performance, with more reported since [48]. Among the genes reported, the angiotensin-1-converting enzyme insertion/deletion (*ACE* I/D) and the α-actinin-3 (*ACTN3*) R577X polymorphisms have been the most extensively studied (see Table 1) and, in general, consistently associated with elite endurance and sprint performance [49, 50]. In a recent review [41], we argue that the limited progress achieved today in the field of sport and exercise genomics is due to limitations in the number of genetic variants studied in small and often heterogeneous cohorts, resulting in “spurious and conflicting results”. There is an evident need for larger collaborative efforts involving clearly defined phenotypes, control of sources of variability, and rigorous replications in order to produce any meaningful results, which has led to the formation of the “Athlome Project Consortium” (www.athlomeconsortium.org). This international collaborative initiative brings together a large databank, expertise and state-of-the-art “omics” technologies from around the world, aiming to understand genetic variation underlying athletic performance, adaptation to exercise training, and injury predisposition. The review by Wang et al. [41] presents the current cohorts and projects involved in the Athlome Consortium and highlight the need for a paradigm shift of the *status quo* to the era of sport and exercise genomics. In particular, an unbiased exploration of the human genome is needed utilising the full power of genomics, epigenomics, and transcriptonomics, in combination with large-scale, replicable study designs [51]. Notable highlights in this regard from the Athlome Consortium is the first published GWAS of athletic performance [52] and the declaration of the sequencing of 1000 of the world’s greatest athletes in the 1000 Athlomes project [53]. Specifically, GWASs were undertaken on 2 cohorts of elite endurance athletes (GENATHLETE and Japanese endurance runners) and their respective controls, from which a panel of 45 candidate single nucleotide polymorphisms (SNPs) was identified, and tested for replication in 7 additional cohorts of endurance athletes and controls from Australia, Ethiopia, Japan, Kenya, Poland, Russia, and Spain. This first of its kind study of elite athletes was based on a total of 1520 endurance athletes (835 of them had competed in World Championships or Olympic Games) and 2760 controls. This initial GWAS attempt failed to identify a panel of genomic variants common to these elite endurance athlete groups due, to the study being underpowered to identify alleles with small effect sizes, and/or due to the use of an earlier generation gene microarray with only 195,000 gene markers (Illumina® CardioMetabochip, Illumina USA), as opposed to some 40 million common polymorphic sites possible, let alone the absence of other genomic features only accessible with full genome sequencing. The 1000 Athlomes Project is therefore timely, as it aims to sequence 1000 genomes of sprinters and distance runners of the highest level from West and East African descent (i.e., world record holders, Olympians and World Champions). It is envisaged that the large amount of genotype data to be generated from the 1000 Athlomes Project will serve as a reference panel for future performance studies and guide other extreme phenotype studies in biomedical science.

Undoubtedly, and as previously outlined, science has evolved significantly throughout the last two centuries, with the early nineteenth century Galtonian model leading to research on heritability of athletic performance via family/twin studies which, in turn, gave rise to studies on gene identification through hypothesis-driven and hypothesis-generating genetic association studies in unrelated individuals. Following the ENCODE (Encyclopedia of DNA Elements) Project (www.encodeproject.org) and the 1000 Genomes Project (www.internationalgenome.org) (Table 1), over 88 million genetic variants have been characterised and over 99% of SNPs reported with a frequency of >1% for a variety of ancestries [54]. As such, the now widely accepted view of human genomics is far more realistic, complex and exciting with *extremely large* but finite numbers of variants with almost infinite possible permutations.

A more complete understanding of the interplay between the molecular basis of elite human performance and the environment will also require deciphering the epigenetic response to environmental stimuli; the changes in gene function that cannot be explained by alterations in the DNA sequence. Several animal and human studies have already provided novel insights into how internal and external environmental factors can influence physiologic processes by regulating gene activity and expression. For example, genome-wide epigenetic changes can be induced by acute and chronic exercise in skeletal muscle [55], adipose tissue [56] and the brain [57, 58]. For example chromatin modifications seem to be involved in triggering the gene expression responses required for physiological and functional adjustments in neurons mediating cognitive processing of stressful events [59]. Epigenetics may therefore be an attractive hypothesis to explain the seemingly paradoxical findings obtained in twin investigations where identical twins differ in some heritable traits for reasons other than the traditional genetic basis of inheritance [60, 61]. Epigenetic differences in genetically identical humans have been demonstrated repeatedly [62], and epigenetic markers appear to be at the interface between environmental stimuli and long-lasting molecular, cellular and behavioral phenotypes [63]. Our knowledge of sport and exercise epigenetics remains limited, and complex mechanisms that modulate gene expression are largely unknown [64]. A great challenge for sport and exercise genomics for the future is to dissect the role of epigenomic alterations in facilitating physiological, metabolic, cognitive, emotional and behavioural changes that empower Olympic athletes to push performance beyond perceived limits.

## Conclusions

While the influence of nature (genes) and nurture (environment) on elite sporting performance remains difficult to precisely determine, the dismissal of either as a contributing factor to performance is unjustified. It is accepted that a complex interaction of a combination of innumerable factors may mold a talented athlete into a champion. In their most basic form, these factors amount to genetics, training and preparation. The contribution of each is absolutely necessary in the making of a world-class athlete. The essential role of practice and training is widely and indisputably recognised. Individual variation, however, in terms of starting performance levels and subsequent response to training (i.e., final performance levels attained with the same amount of training), clearly illustrate the prominent role of genetic factors and their interaction with training and the environment. The overwhelming and accumulating evidence, amounted through empirical and experimental research spanning over almost two centuries, tips the balance in favour of nature in the “nature” and “nurture” debate. In other words, truly elite-level athletes are built – but only from those born with innate ability. This conclusion is in line with the prophetic text by Galton, some 150 years ago who wrote “there is nothing in what I am about say that shall underrate the sterling value of nurture, including all kinds of sanitary improvements; may, I wish to claim them as powerful auxiliaries to my cause; nevertheless, I look upon race as far more important than nurture.” [65].
